# Thrombosis, Neuroinflammation, and Poststroke Infection: The Multifaceted Role of Neutrophils in Stroke

**DOI:** 10.1155/2017/5140679

**Published:** 2017-02-26

**Authors:** Johanna Ruhnau, Juliane Schulze, Alexander Dressel, Antje Vogelgesang

**Affiliations:** ^1^Department of Neurology, University Medicine Greifswald, Greifswald, Germany; ^2^Department of Neurology, Carl-Thiem-Klinikum, Cottbus, Germany

## Abstract

Immune cells can significantly predict and affect the clinical outcome of stroke. In particular, the neutrophil-to-lymphocyte ratio was shown to predict hemorrhagic transformation and the clinical outcome of stroke; however, the immunological mechanisms underlying these effects are poorly understood. Neutrophils are the first cells to invade injured tissue following focal brain ischemia. In these conditions, their proinflammatory properties enhance tissue damage and may promote ischemic incidences by inducing thrombus formation. Therefore, they constitute a potential target for therapeutic approaches and prevention of stroke. Indeed, in animal models of focal brain ischemia, neutrophils have been targeted with successful results. However, even in brain lesions, neutrophils also exert beneficial effects, because they are involved in triggering immunological removal of cell debris. Furthermore, intact neutrophil function is essential for maintaining immunological defense against bacterial infections. Several studies have demonstrated that stroke-derived neutrophils displayed impaired bacterial defense capacity. Because infections are known to impair the clinical course of stroke, therapeutic interventions that target neutrophils should preserve or even restore their function outside the central nervous system (CNS). This complex situation requires well-tailored therapeutic approaches that can effectively tackle immune cell invasion in the brain but avoid increasing poststroke infections.

## 1. Introduction

Stroke is one of the leading causes of death in the world. Most stroke-related deaths result from thrombotic occlusion of brain vessels. Infections are a known risk factor for acute stroke [1]. This enhanced risk is at least in part due to activated immune cells that interact with platelets and release coagulation factors, which amplify thrombus formation. In this review, Section 2 describes the deleterious role of neutrophils in initiating thrombosis. Stroke treatment has been limited to a strategy of rapid revascularisation, initiated within 4.5 h of onset, by inducing thrombolysis with recombinant tissue plasminogen activator (rtPA). In the setting of intracranial large artery occlusion (iLAO) this treatment is associated with low rates of recanalization and high rates of neurological morbidity and severe disability. Here endovascular therapy, particularly mechanical thrombectomy, is a promising therapeutic adjunct to rtPA [2]. The early multicentre trails IMS III [3], MR RESCUE [4], and SYNTHESIS Expansion [5] failed to show a benefit from endovascular intervention. However, quite recently, a series of studies with improved protocols demonstrated that mechanical recanalization in combination with rtPA administration was a superior treatment strategy in patients with iLAO compared to rtPA treatment alone [6–11].

The control of inflammation at the site of the ischemic lesion is a potential therapeutic target that has resulted in promising results in experimental stroke studies and may allow for a longer therapeutic window. Cellular invasion and the resulting proinflammatory response develop within days rather than hours and contribute to secondary lesion growth, which enhances ischemic brain tissue destruction. The role of neutrophils in these central events and corresponding therapeutic approaches are discussed in Section 3.

**Figure 1:**
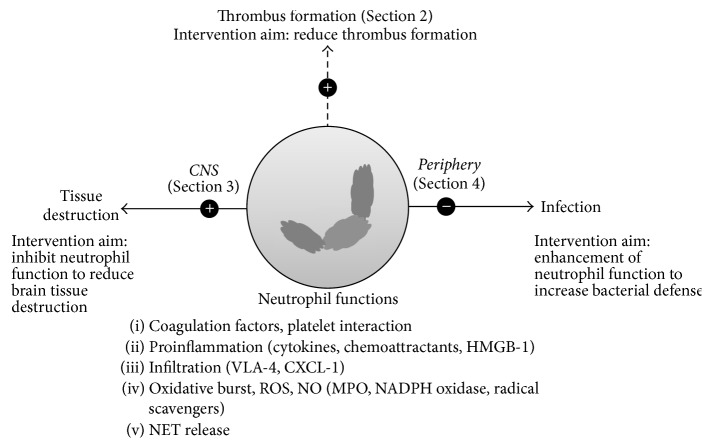
Neutrophil functions that can be targeted to reduce brain tissue destruction after stroke. Targets include factors involved in proinflammation, infiltration of immune cells, production of reactive oxygen species (ROS) and nitric oxide (NO), enzymatic functions of myeloperoxidase (MPO) and nicotinamide adenine dinucleotide phosphate (NADPH) oxidase, and the release of neutrophil extracellular trap (NET) components. Inhibiting these pathways may also reduce thrombus formation and prevent recurrent stroke. In addition, after a stroke, patients undergo poststroke immune suppression, which includes impaired oxidative burst and NET formation, induced by catecholamines. Enhancing bacterial defense by targeting these mechanisms could decrease the risk of secondary infections. Therefore, poststroke immune modulation must take into account the fact that immune suppression has opposing effects in the central nervous system and in the periphery. HMGB-1: high mobility group protein box 1; VLA-4: very-late-antigen 4; CXCL-1: chemokine (C-X-C motif) ligand 1.

Another aspect of stroke is the systemic immune suppression that predisposes patients to systemic bacterial infections. Infection by itself is an independent risk factor of an unfavorable outcome in ischemic stroke [12, 13]. Infections contribute to worse clinical outcome measures, increased risk of recurrent stroke, and death. We discuss the function of neutrophils in bacterial defense, after stroke, and the associated therapeutic targets in Section 4.  Figure 1 summarizes the aspects of stroke and the therapeutic targets evaluated in this review.

## 2. Neutrophils Promote Thrombosis

Neutrophils promote thrombus formation through different mechanisms, including the release of molecules involved in neutrophil extracellular trap (NET) formation, the release of proteases, and direct interactions with platelets. This prothrombotic process might (i) increase the risk of ischemic stroke and (ii) promote further thrombosis during acute stroke. The percentage of neutrophil-platelet interactions was enhanced in patients with symptomatic carotid stenosis [14]. However, the formation of leukocyte-platelet aggregates was reduced by inhibiting GPIIb/IIIa and selectin adhesion molecules. Leukocyte-platelet aggregate formation after ischemic stroke and reperfusion may be a useful biomarker and potential therapeutic target, since these aggregates also promote intravascular thrombus formation [15].

Neutrophils adhere to injured vessels immediately, preceding platelets, by binding to the activated endothelium through an interaction between leukocyte function-associated antigen and ICAM-1. This is an important step for the activation and accumulation of thrombocytes, and blocking this step might be an efficient strategy for reducing cerebral thrombosis. Both candesartan and dipyridamole were found to inhibit the adhesion of neutrophils to vascular endothelium in patients with ischemic stroke, but not in patients with chronic stroke or healthy individuals [16].

The protease, cathepsin G, is released by neutrophils and acts on coagulation factors to promote clot formation. Inhibition of cathepsin G decreased thrombus formation and reduced brain injury. The result was an improvement in the neurobehavioral outcome in a mouse model of ischemic stroke [17]. On the other hand, neutrophils also release the protease ADAMTS13, which cleaves hyperactive ultralarge von-Willebrand-factor and, thus, reduces acute cerebral inflammation after ischemic stroke [18].

An additional neutrophil contribution to thrombus formation results from the prothrombotic activity of NETs [19]. NETs have a web-like structure composed of DNA, histones, and specific granule proteins, such as neutrophil elastase and MPO, which can be released in response to various stimuli. Their primary function is to trap bacteria and exert bactericidal effects [20]. Platelets can bind to released NETs and get activated by histones [19, 21]. Interaction with neutrophils takes place through P-selectin and neutrophil P-selectin glycoprotein ligand-1 [22]. Upon activation platelets also express HMGB-1 and expose it on their surface promoting additional NET release by neutrophils [23], a self-energizing process that even activates the extrinsic coagulation pathway and may be responsible for further thromboinflammation observed after stroke [24]. Administration of DNase I resulted in the resolution of NETs. This strategy had a protective in vivo effect, in murine models of ischemic stroke [25].

## 3. Inflammatory Role of Neutrophils within the Brain

Necrotic cell death within the infarcted area leads to the release of proinflammatory cytokines and a rapid infiltration of immune cells. Neutrophils are the first cells recruited into the brain within minutes after stroke. The mechanism of neutrophil entry into the brain after stroke was investigated in permanent and transient experimental stroke models with in vivo imaging. Blood-borne neutrophils immediately migrate, even against blood flow, and then transmigrate out of blood vessels to reach the injured brain area [26]. The zenith of neutrophil invasion is reached between 48 and 72 h after stroke [27]. In physiological conditions, the blood brain barrier (BBB) controls the entry of immune cells into the brain. However, neutrophil entry is facilitated by a local BBB breakdown induced by ischemia [28].

The impact of immune cell invasion is a controversial issue. Although these cells might play a role in the initiation of tissue repair, their detrimental effects dominate. This was demonstrated in experimental stroke settings, where invading neutrophils enhanced ischemic neurotoxicity through different actions [29]. When neutrophils are activated, they produce reactive oxygen species (ROS), like superoxide radicals and hydrogen peroxide. In addition, they release enzymes from different granules, like cathepsin G, collagenase, gelatinase, and heparinase, which contribute to ROS-mediated extracellular matrix breakdown and vascular damage. As described in Section 2 neutrophils can activate complement and release cell content, like DNA, during suicidal extracellular trap formation. This antibacterial defense mechanism also includes the release of neutrophil elastase, which was shown to increase vascular permeability [30–32]. In addition, neutrophil release of proinflammatory mediators initiates a self-energizing cascade of proinflammation and destruction. Resident microglia can fight this detrimental destruction to a minor extent, by engulfing neutrophils [29].

These detrimental effects of neutrophils make them a prime target in novel therapies for stroke. Indeed, in experimental focal brain ischemia models, a variety of therapeutic interventions successfully reduced lesion size. One approach was to block proinflammatory cytokines and mediators that act as chemoattractants. For example, antagonization of C-X-C motif chemokine receptor 2 (CXCR-2) prevented recruitment of neutrophils to the infarct area [33]. Another neutrophil chemoattractant, chemokine (C-X-C motif) ligand 1 (CXCL-1), is induced by interleukin 17 (IL-17), which is released by *γδ* T-cells. Blocking this pathway with an anti-IL-17-antibody reduced the experimental stroke lesion size [34]. In addition, neutrophil extravasation was shown to be mediated by very-late-antigen 4 (VLA-4) in an experimental stroke model. Accordingly, blocking VLA-4 reduced lesion size [26].

A different approach, which does not interfere with neutrophil invasion, is to block the neutrophil proinflammatory function. Oxidative stress, caused by an overload of ROS, contributes to various acute, chronic, and inflammatory diseases. Thus, this mechanism has been suggested as a target of stroke therapy. In the preclinical setting, beneficial effects were achieved by inhibiting type 4 nicotinamide adenine dinucleotide phosphate (NADPH) oxidase (NOX4). In experimental stroke models, brain damage was also ameliorated by inhibiting myeloperoxidase oxidant (MPO) production, with N-acetyl lysyltyrosylcysteine amide or with the flavonoid, eriodictyol [35, 36]. Moreover, neutrophil infiltration, assessed by measuring MPO activity, and infarct volume were significantly reduced following the administration of AM-36 (1-(2-(4-chlorophenyl)-2-hydroxy)ethyl-4-(3,5-bis-(1,1dimethylethyl)-4-hydroxyphenyl) methylpiperazine). This arylalkylpiperazine is a neuroprotectant with combined antioxidant and Na(+) channel-blocking actions [37].

Nitric oxide (NO) produced by inducible NO synthase (iNOS) contributes to ischemic brain injury. iNOS expression is predominantly found in invading neutrophils after stroke. When iNOS(+/+), but not iNOS(−/−), neutrophils were transferred into iNOS(−/−) mice, infarct volume increased. That result identified iNOS as an important mediator of secondary tissue destruction [38]. The inhibition of oxidative radical production was reported to be a favorable strategy in lacunar infarctions [39]. In contrast, the administration of Edaravone, a free radical scavenger, in patients with cardiogenic embolism increased hemorrhagic transformation [40]. In patients receiving rtPA treatment hemorrhagic complications and particular symptomatic intracerebral hemorrhage are more frequent in blacks and Asians. Since Mehta et al. administered Edaravone to patients with an Asian background it is possible that the higher bleeding rate was caused by ethnic-related reasons [41].

Uric acid, another free radical scavenger, was thought to protect the brain from oxidative injury. Until now studies investigating the neuroprotective effect of UA after stroke remain controversial [42]. While descriptive studies find that higher concentrations of UA in serum are beneficial in patients with stroke treated by thrombolysis [43, 44] the results of the URICO-ICTUS (study of intravenous uric acid administered during alteplase treatment for ischemic stroke) showed only a beneficial outcome for selected patient groups, for example, women [45].

Nevertheless it is known that different additional factors like advanced age, increased time to treatment, the extent of ischemic injury prior to administration of therapy, higher baseline National Institutes of Health Stroke Scale (NIHSS) score, high systolic blood pressure, or diabetes mellitus increase the risk of hemorrhagic incidence after stroke [46]. Therefore, treatment options might depend on the combination of individual factors.

Another molecule discussed in the modulation of poststroke immune response is the HMGB-1. This DNA-binding protein is passively released during stroke from cells undergoing necrosis. This damage-associated molecular pattern molecule can also be actively secreted by immune cells and is released and exposed by platelets promoting thrombus formation as described in Section 2. In clinical studies, elevated plasma HMGB-1 levels were detected in patients with acute ischemic stroke. A correlation between HMGB-1 levels and circulating leukocytes was verified [47]. It was also shown that HMGB-1 contributed to tissue destruction by recruiting neutrophils and inducing extracellular trap formation [48, 49]. Reductions in plasma HMGB-1 levels with cannabinoids were associated with reductions in infarct size and in the number of activated neutrophils [50]. The rapid early changes observed in experimental stroke models could be prevented by blocking *β*-adrenoceptors with propranolol or by neutralizing HMGB-1 activity with antibodies or an antagonist of its receptor, the receptor for advanced glycation end-products (RAGE) [51, 52]. These treatments were applied before and directly after stroke induction. To the best of our knowledge, no studies have reported delayed treatment regimens. Therefore, it remains unknown how the timing of catecholamine and HMGB-1 actions affects the development of stroke-induced immune alterations.

In addition to enhancing ischemic injury and the subsequent signaling cascades, neutrophils are also involved in reperfusion injury. Risk of hemorrhagic transformation is increased by as much as tenfold after intravenous rtPA administration, largely due to reperfusion injury and the toxic effects of rtPA [27]. High neutrophil counts and a high neutrophil-to-lymphocyte ratio were independently associated with worse outcomes at 3 months, in patients with stroke that were treated with rtPA [53, 54]. Similar results were found for patients with intracerebral hemorrhage [55]. Interestingly, treatment with rtPA induced neutrophil degranulation in vitro. In a cohort of 60 patients that underwent thrombolysis, during the first hours after drug administration, a peak of neutrophil degranulation products was observed, including matrix metalloproteinase- (MMP-) 9, MMP-8, neutrophil elastase, and MPO. Even though tissue destruction by neutrophils seems more pivotal, also protective molecules like tissue inhibitor of metalloproteinase- (TIMP-) 1 and TIMP-2 are elevated in serum [56].

Granulocyte colony stimulating factor (G-CSF) had a neuroprotective effect in several models of experimental stroke. Administration of G-CSF decreased infarct size and improved motor function recovery [57]. A recent meta-analysis of several small clinical trials concludes, though, that G-CSF did not improve stroke outcome in patients suffering from stroke [58]. In experimental stroke models applying rtPA, no beneficial effects of additional G-CSF administration were observed; instead an increased risk of hemorrhage occurred within the infarct area at 72 h after stroke [59]. In these models neutrophil blood counts were increased and neutrophilic activation occurred within 15 min after reperfusion, and it remained evident after 24 h [60]. Neutrophils might be mediators of hemorrhagic complications after thrombolysis; thus, they could represent new targets for neuroprotective strategies in patients treated with rtPA.

## 4. Anti-Infective Role of Neutrophils in the Periphery

Even before the appearance of stroke, neutrophils might play a role as a predictive marker of stroke risk. The neutrophil-to-lymphocyte ratio (NLR) was directly associated with the risk of stroke in patients with atrial fibrillation. As a predictor of stroke, the NLR appeared to be important for refining the risk of stroke and for improving the management of patients with atrial fibrillation and a low CHA2DS2-VASc score, a score for atrial fibrillation stroke risk [61]. The NLR was also increased in symptomatic intermediate carotid artery stenosis. It was shown that an elevated NLR was an independent variable associated with carotid artery plaques becoming symptomatic [62, 63].

In peripheral blood of patients with stroke, lymphocyte number and HLA-DR expression on monocytes decline, but neutrophil numbers increase. Animal studies have described an immediate reduction in spleen volume following cerebral ischemia. The change due to cerebral ischemia in human spleens was described as a biphasic process; splenic volumes initially decreased over time, reached a nadir at 48 h after stroke onset, and then increased thereafter. This process was positively correlated to the percentage of peripheral blood neutrophils [64]. Additionally, experimental stroke led to an activation of the hematopoietic system, via increased stimulation of the autonomic nervous system. This stimulation resulted in increased hematopoiesis and greater output of neutrophils from the bone marrow [65].

As previously discussed, despite an increase in granulocyte numbers, infections can occur in patients within days after a stroke [66]. A diagnosis of infection after stroke can be very difficult, because the hallmark signs, like fever and inflammation, can be present in patients as a consequence of neurological damage that disrupts homeostatic regulation of body temperature [67]. A method for identifying patients prone to subsequent infection could promote early interventions that reduce poststroke bacterial burden and improve clinical outcome. However, powerful prognostic biomarkers remain to be identified.

The role of neutrophils in infections after stroke is controversial. In the Enlimomab study protocol, patients with stroke were treated with anti-ICAM-1 antibodies to diminish neutrophils; however, those patients experienced an increased rate of infection, particularly pneumonia on day 5 after Enlimomab administration (2.2% versus 1.6% in placebo patients) as serious adverse event. Moreover, the infections were associated with a worse outcome [68]. In that study, neutrophils seemed to be important preventers of poststroke infections; inhibiting neutrophils with neutrophil inhibitory factors in humans did not increase the rate of infections, but this intervention neither reduced infarct volume nor improved stroke outcome [69].

Neutrophils obtained from patients that underwent neurosurgical interventions for hemorrhagic stroke showed significantly lower levels of oxygen species than neutrophils from healthy controls [70]. An earlier report suggested that alterations in neutrophil function occurred in patients with stroke, and these alterations were indicated by measurements of the granulocyte antisedimentation rate [71]. In ischemic stroke, ROS was impaired in monocytes and granulocytes, but no alterations were found in phagocytosis, migration, or the amounts of human neutrophil peptides 1 to 3 (HNP 1–3). However, patients with infections after stroke showed lower amounts of ROS than patients without a poststoke infection. Therefore, phagocyte dysfunction seemed to be associated with stroke-associated infections [72].

In patients with stroke, NETs were impaired in the early phase of stroke (day 1 of admission) and recovered function on day 5 after admission. Because these phagocyte dysfunctions were present upon admission to the stroke unit, they might contribute to the susceptibility to stroke-associated infections [72].

According to our current understanding of stroke-induced immune alterations, immediately after a stroke, a “storm” of stress hormones, particularly catecholamines, are released by the adrenal gland and via direct sympathetic innervation of the lymphoid organs (reviewed in [13, 73, 74]). This concept was derived from the original observation that a *β*-blockade at the time of stroke could reverse most effects of stroke-induced immune alterations observed in an experimental stroke model [51]. It is currently known that neutrophils express different receptors that are regulated by glucocorticoids and catecholamines. Interestingly, in vitro experiments have attributed stroke-induced neutrophil impairments to the influence of catecholamines [72].

## 5. Conclusions

Neutrophils are a promising target in stroke therapy. However, the development of novel, neutrophil-based therapies must take into account the opposing effects that immune suppression has in the CNS and in the periphery. Although bacterial defense must be maintained or enhanced in the periphery, the immune response in the brain is largely detrimental and should be inhibited. This quandary has been reflected in clinical trial results that could not reproduce preclinical experimental successes.
